# Porcine circovirus type 2 activates PI3K/Akt and p38 MAPK pathways to promote interleukin-10 production in macrophages via Cap interaction of gC1qR

**DOI:** 10.18632/oncotarget.7362

**Published:** 2016-02-13

**Authors:** Qian Du, Yong Huang, Tongtong Wang, Xiujuan Zhang, Yu Chen, Beibei Cui, Delong Li, Xiaomin Zhao, Wenlong Zhang, Lingling Chang, Dewen Tong

**Affiliations:** ^1^ College of Veterinary Medicine, Northwest A&F University, Xianyang, Shaanxi, P. R. China

**Keywords:** porcine circovirus type 2, capsid, IL-10, gC1qR, Immunology and Microbiology Section, Immune response, Immunity

## Abstract

Porcine circovirus type 2 (PCV2) infection caused PCV2-associated diseases (PCVAD) is one of the major emerging immunosuppression diseases in pig industry. In this study, we investigated how PCV2 inoculation increases interleukin (IL)-10 expression in porcine alveolar macrophages (PAMs). PCV2 inoculation significantly upregulated IL-10 expression compared with PCV1. Upon initial PCV2 inoculation, PI3K/Akt cooperated with NF-κB pathways to promote IL-10 transcription via p50, CREB and Ap1 transcription factors, whereas inhibition of PI3K/Akt activation blocked Ap1 and CREB binding to the *il10* promoter, and decreased the binding level of NF-κB1 p50 with *il10* promoter, leading to great reduction in early IL-10 transcription. In the later phase of inoculation, PCV2 further activated p38 MAPK and ERK pathways to enhance IL-10 production by promoting Sp1 binding to the *il10* promoter. For PCV2-induced IL-10 production in macrophages, PCV2 capsid protein Cap, but not the replicase Rep or ORF3, was the critical component. Cap activated PI3K/Akt, p38 MAPK, and ERK signaling pathways to enhance IL-10 expression. In the whole process, gC1qR mediated PCV2-induced PI3K/Akt and p38 MAPK activation to enhance IL-10 induction by interaction with Cap. Depletion of gC1qR blocked PI3K/Akt and p38 MAPK activation, resulting in significant decrease in IL-10 production in PCV2-inoculated cells. Thus, gC1qR might be a critical functional receptor for PCV2-induced IL-10 production. Taken together, these data demonstrated that Cap protein binding with host gC1qR induction of PI3K/Akt and p38 MAPK signalings activation is a critical process in enhancing PCV2-induced IL-10 production in porcine alveolar macrophages.

## INTRODUCTION

Interleukin-10 (IL-10), an important immunomodulatory cytokine, has attracted much attention because of its anti-inflammatory and immunosuppressive properties. As a major immunosuppressive cytokine, IL-10 can inhibit the production of various pro-inflammatory cytokines, such as IL-12, IL-6 and TNF-α, *et al*, and also inhibit cell-mediated immune responses and antigen presentation [1]. Monocytes/macrophages, dendritic cells and B cells appear to be the predominant cell types secreting IL-10 following virus infection [2]. IL-10 expression can be highly regulated at both transcriptional and post-transcriptional levels through different molecular mechanisms upon different kind of stimuli. In monocytes/macrophages, nuclear factor-kappa B (NF-κB) p50/p50 complexes have been proved to directly regulate the transcription of IL-10 by binding with *il10* promoter [2, 3]. In additional, mitogen-activated protein kinases (MAPKs), ERK and p38 MAPK signaling, are also involved in the transcriptional regulation of IL-10 in macrophages and DCs employed as important transcriptional co-regulation signalings [2]. In addition, phosphoinositide 3 kinase (PI3K)/Akt pathway also modulates IL-10 production by blocking glycogen synthase kinase 3 (GSK3), which prevents the binding of activator protein 1 (AP1) to the *il10* promoter [4]. IL-10 has been proved to be up-regulated and play key roles in the immunosuppression-associated immunopathologic alterations in some virus infectious diseases [5–8]. Specially, PCV2 and porcine reproductive and respiratory syndrome virus (PRRSV) have been demonstrated to induce immunosuppression by upregulation of IL-10 expression [5, 8].

PCV2 is a major pathogen to cause emerging viral infectious disease in world pig husbandry [9]. PCV2 belongs to the family *Circoviridae* which is considered to originate from a plant nanovirus [10]. Besides piglets, mice and calves are also found to be able to infect this virus [11, 12]. In human, this virus has been reported to be detected in sera [13], but no antibodies against PCV2 are detected in sera [14]. PCV2 infected piglets are easily to develop concomitant infection, such as PRRSV, porcine parvovirus (PPV) and *haemophilus parasuis*, suggesting that PCV2 associated diseases (PCVAD) is actually an immunosuppressive disease. Previous studies have revealed that PCV2 could upregulate IL-10 production both *in vivo* and *in vitro* [8, 15]. However, how PCV2 induce these cells to produce IL-10 is still largely unknown.

PCV2 genome contains 11 putative open reading frames (ORFs), but only four of them are described to encode proteins. ORF1 encodes the replicase (Rep) of the virus, which is considered to be the essential protein for viral replication [16]. ORF2 encodes the capsid protein (Cap), which is the only structural protein of PCV2 and includes a nuclear location signal in the N-terminate [17]. The protein encoded by ORF3 is a non-structural protein that has been reported to associate with viral replication and pathogenesis [18], and lately found to promote secretion of IL-6 and IL-8 in porcine epithelial cells [19]. The ORF4 encoding protein is latterly identified to play a role in suppressing PCV2-induced apoptosis, and identified as nonessential component for PCV2 replication [20]. In addition, the DNA and CpG oligonucleotides (CpG-ODN) from PCV2 are found to be able to suppress IFN-α secretion in peripheral blood mononuclear cells (PBMCs) and bone marrow-derived dendritic cells (BMDCs) and have indicated to be main component for activation of NF-κB signaling pathway [21, 22]. Up to date, however, which component of PCV2 to play central role in PCV2-induced IL-10 secretion is still unclear. Numerous cellular proteins have been identified as the binding proteins of PCV2 Rep, Cap or ORF3 proteins, including transcriptional regulator *c-myc*, zinc finger protein 265 (ZNF265), thymine DNA glycosylase, E3 ubiquitin ligase family member MKRN1 and pPirh2, C1q receptor gC1qR, chaperonin Hsp40, and a porcine regulator of G protein signaling related to human RGS16, poRGS16 [23, 24]. However, the biological effect of these viral and cellular protein interactions remains largely unknown.

In the present study, we firstly investigated the activation characteristics of NF-κB, PI3K/Akt, ERK and p38 MAPK signaling pathways in PCV2-inoculated PAMs and the roles of these pathways in PCV2-induced IL-10 expression. Then, we identified which component of PCV2 and their binding cellular proteins played the central role in PCV2-induced IL-10 secretion in PAMs. Notably, in this study, we found PCV2 inoculation activated variant signalings in different characteristics to induce and enhance IL-10 transcription in PAMs, and gC1qR protein, a C1q binding protein, was firstly found to play a pivotal role in PCV2 induction of PI3K/Akt and p38 MAPK activation and IL-10 expression via binding with the Cap protein of PCV2.

## RESULTS

### Kinetics of IL-10 expression in PCV2-inoculated PAMs

**Figure 1:**
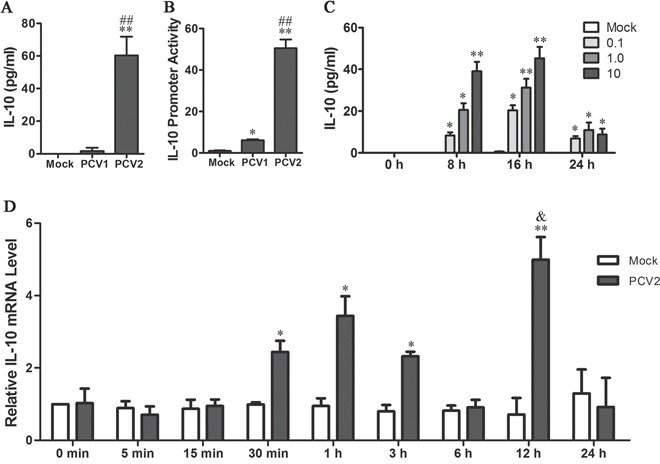
PCV2 induces IL-10 production in porcine alveolar macrophages (PAMs) **A.** IL-10 secretion was measured by ELISA in the primary PAMs (1×10^6^ cells) inoculated with 1 MOI of PCV1 or PCV2 for 24 h. **B.** The activities of *il10* promoter were measured in the PAMs inoculated with 1 MOI of PCV1 or PCV2 for 24 h via Dual-Luciferase Reporter Assay System. **C.** The kinetic of IL-10 production in PCV2-inoculated PAMs (1×10^6^ cells) at 0.1, 1 or 10 of MOI. The supernatants were harvested at each 8 h to measure IL-10 secretion by ELISA. **D.** The IL-10 mRNA levels of the PAMs inoculated with 1 MOI PCV2 were detected by quantitative PCR at the indicated times post-inoculation. Mock infection was measured at the same time. The results are mean ± SEM of 3 independent experiments. *P < 0.05, **P < 0.01 versus Mock infected cells at the same times. ^##^*P* < 0.01 versus PCV1-inoculated cells (A, B). ^&^*P* < 0.05 versus PCV2-inoculated cells at 1 h (D).

Previous *in vivo* and *in vitro* studies have revealed that PCV2 infection induces porcine IL-10 expression in spleens, lymph nodes and thymus in infected pigs, and cultured monocytes and dendritic cells, whereas PCV1 was not reported to induce IL-10 production *in vivo* or *in vitro* studies [8, 15]. We therefore firstly investigated the difference between PCV1 and PCV2 in induction of IL-10 expression. ELISA detection showed that PCV2 inoculation significantly induced IL-10 secretion in primary PAMs and porcine alveolar macrophages cell line (3D4/21) cells at 24 hours post inoculation (h p.i.), whereas PCV1 inoculation barely induced detectable IL-10 secretion in primary PAMs and 3D4/21 cells (Figure 1A and Supplemental Figure 1). Consistent with the change of IL-10 protein levels, IL-10 transcriptional levels in PCV2-inoculated cells were about 8.3-fold higher than that in PCV1-inoculated cells in luciferase reporter assays (Figure 1B), suggesting that PCV2 possesses a stronger ability than PCV1 to promote IL-10 expression in PAMs.

Next, we examined the kinetic of IL-10 production in PAMs response to different titers of PCV2 inoculation by ELISA assay. A detailed time-course showed that, after PCV2 inoculated PAMs at a multiplicity of infection (MOI) of 0.1, 1 or 10, IL-10 secretion greatly increased in the first 8 hours, and reach in highest levels in the second 8 hours, then declined in third 8 hours (Figure 1C). At 1 MOI of PCV2 inoculation, the levels of IL-10 mRNA appeared to be increased at 0.5-3 h p.i. firstly, subsequently declined, and then secondarily increased at 12 h p.i. (Figure 1D). Notably, the secondary increase of IL-10 mRNA levels was higher than the first increase upon PCV2 inoculation (Figure 1D). Meanwhile, no viral progeny were detected in the culture media within 24 hours upon PCV2 inoculation (data not shown). These results suggested that *il10* gene was induced to be transcripted for twice upon PCV2 inoculation, and that there might be some different regulation involved in twice induction.

### PCV2 inoculation activates NF-κB, PI3K/Akt and MAPK signalings to promote IL-10 expression in macrophage

**Figure 2:**
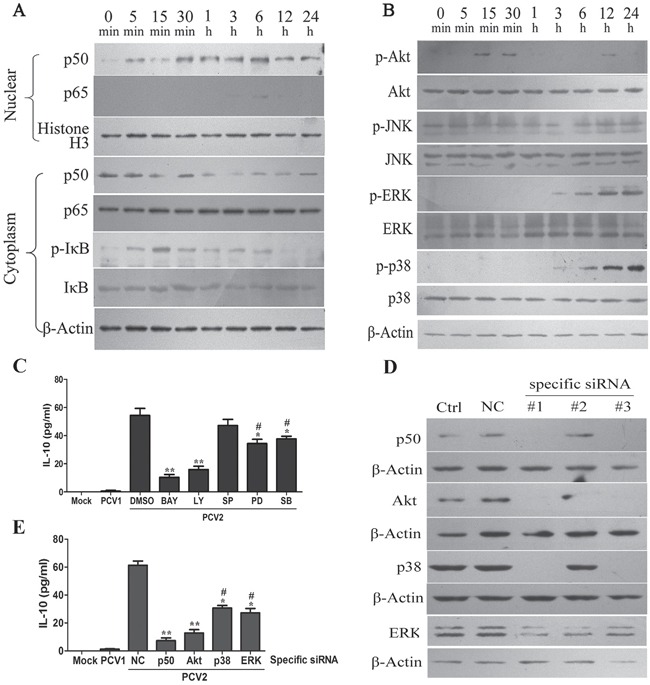
PCV2 inoculation induces IL-10 expression via activating NF-κB, PI3K/Akt, ERK and p38 MAPK signaling pathways in PAMs **A.** The distribution of NF-κB p50 and p65, and the levels of p-IκB and IκB in the PAMs inoculated with 1 MOI PCV2 were detected by western blotting at indicated times post-inoculation. **B.** The levels of Akt, JNK, ERK, p38 MAPK and their phosphorylation in the PAMs inoculated with 1 MOI of PCV2 were detected by western blotting at indicated times post-inoculation. **C.** The effects of inhibiting NF-κB p50, Akt, ERK and p38 MAPK pathways on the PCV2-induced IL-10. The specific inhibitors of NF-κB (BAY11-7082, 5 μM), PI3K/Akt (LY249002, 10 μM), JNK (SP600125, 10 μM), ERK (PD98059, 20 μM) and p38 MAPK (SB203580, 10 μM) treated cells at 1 h pre-infection, and then cells were infected with 1 MOI of PCV2 for 24 h. The IL-10 secretions were detected by ELISA. **D.** The efficiency of the siRNAs were evaluated by western blotting after the specific siRNA for NF-κB p50, Akt, p38 and ERK were transfected to cells for 48 h, respectively. **E.** The effects of downregulating NF-κB p50 (#1), Akt (#1), ERK (#1) and p38 MAPK (#1) on the IL-10 production in PCV2-inoculated PAMs. The most efficiently specific siRNAs or negative control (NC) siRNA transfected PAMs (1×10^6^ cells) for 24 h, and PCV2 infected the cells for another 24 h. The IL-10 secretion of each cells were analyzed by ELISA. The data shown are representative of three independent experiments. *P < 0.05, **P < 0.01 versus DMSO-treated cells (C) or negative control (NC) siRNA-transfected cells (E). ^#^*P* < 0.05 versus LY-treated cells (C) or Akt specific siRNA-transfected cells (E).

NF-κB, PI3K/Akt and MAPKs signalings have been demonstrated to regulate IL-10 production in macrophages [2]. To identify which signaling pathways were involved in the regulation of IL-10 expression in PCV2-inoculated cells, the activities of these signalings and characteristics of activation were identified in PAMs infected with 1 MOI of PCV2. PCV2 inoculation triggered phosphorylation of IκB, which was detectable at 5 min p.i., increased and peaked at 15 min p.i., then declined but maintained in a detectable level to 6 h p.i., while the total IκB were barely changed in the whole process of PCV2 inoculation (Figure 2A). In consistent with phosphorylation of IκB, NF-κB1 p50 unit nuclear translocation greatly increased at 30 minutes post infection and maintained relatively higher level by 24 h.p.i. while NF-κB p65 unit nuclear translocation just showed a slight increase at 6 h post-infection (Figure 2A). These results suggest PCV2 inoculation activates the NF-κB1 p50 signaling in PAMs.

PI3K/Akt and MAPK signalings have been reported to be activated in PCV2 infected PK-15 cells [25-27]. In PCV2-inoculated PAMs, phospho-Akt was detectable as early as 15 min p.i., and rapidly increased from 15 min through to 30 min, followed by a decrease at 1, 3 and 6 h, then increased secondarily at 12 h p.i. (Figure 2B). Whereas phospho-ERK and phospho-p38 were observed till 3 h, increased at 6 h p.i., and maintained up to 24 h p.i. (Figure 2B). However, the phosphorylation levels of the main isoforms of JNK, p46/JNK1 and p54/JNK2 did not appear obvious increase during the infection period (Figure 2B).

Next, we explored the roles of these signalings activation in PCV2-induced IL-10 production. As predicted, addition of NF-κB inhibitor (BAY11-7082), PI3K/Akt inhibitor (LY249002), p38 MAPK inhibitor (SB203580) or ERK inhibitor (PD98059) could significantly attenuate IL-10 accumulation in PCV2-inoculated PAMs (Figure 2C). Consistent with the effects of inhibitors on PCV2-induced IL-10, downregulation of NF-κB1 p50, Akt, p38 MAPK and ERK1/2 by specific siRNAs (Figure 2D) also showed similar effects and characteristics on PCV2-induced IL-10 production (Figure 2E). Notably, IL-10 reduction were more significant in the cells incubated with NF-κB and PI3K/Akt inhibitors or their specific siRNAs than that in the cells incubated with p38 MAPK and ERK inhibitors or their specific siRNAs (Figure 2C and 2E). These results suggested that for PCV2-induced IL-10 expression, the activation of NF-κB and PI3K/Akt pathways play more important roles compared with the activation of p38 MAPK and ERK pathways.

### PI3K/Akt cooperates with NF-κB pathways to promote IL-10 transcription via p50, CREB and Ap1 transcription factors in the earlier phase of PCV2 inoculation

**Figure 3:**
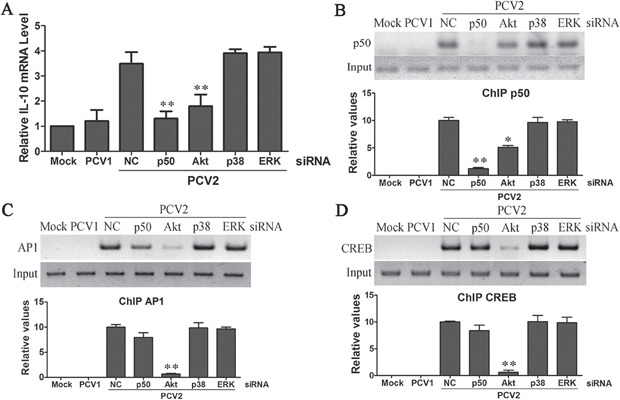
PCV2 inoculation employed PI3K/Akt cooperating with NF-κB pathways to promote IL-10 transcription via p50, CREB and Ap1 transcription factors in the earlier phase PAMs were transfected with the specific siRNAs of NF-κB, Akt, ERK, p38 MAPK, or negative control siRNA for 24 h, then cells were inoculated with 1 MOI of PCV2 for 1 h. The levels of IL-10 mRNA were detected by Q-PCR **A.** At the same time, the cells nuclear fraction were isolation, and the binding activities of NF-κB p50 **B.** Ap1 **C.** and CREB **D.** were detected by ChIP assay. The relative binding levels of transcriptional factor with *il10* promoter were further exactly evaluated by Q-PCR (bottom panel in B, C, D). Mock and PCV1 inoculated cells were used as control in these assays. The results are mean ± SEM of 3 independent experiments. *P < 0.05, **P < 0.01 versus negative control (NC) siRNA-transfected cells.

Q-PCR detection of early IL-10 transcriptional levels showed that down-regulation of NF-κB and Akt markedly attenuated PCV2-induced IL-10 transcription, whereas down-regulation of p38 MAPK and ERK did not inhibit PCV2-induced IL-10 transcription (Figure 3A). Past studies have demonstrated that NF-κB1 p50, Ap1, CREB, Sp1 and Stat3 are major transcriptional factors in NF-κB, PI3K/Akt and MAPKs signalings induction of IL-10 gene expression [2]. To investigate the roles of these transcriptional factors in induction of IL-10 transcription in earlier stage, we examined the binding levels of Ap1, CREB and NF-κB1 p50 to the *il10* promoter in situ within live cells by ChIP assays. Control (mock infection) cells virtually exhibited no binding of either Ap1, CREB or NF-κB1 p50 to the *il10* promoter (Figures 3B-3D). Similarly, PCV1 that did not induce early IL-10 transcription (Figure 3A) also failed to result in these transcriptional factors binding to the *il10* promoter (Figures 3B-3D). However, PCV2 could induce the efficient binding of Ap1, CREB and NF-κB1 p50 to the *il10* promoter (Figures 3B-3D). Furthermore, down-regulation of PI3K/Akt activation with Akt specific siRNA inhibited IL-10 induction, largely blocked transcriptional factor Ap1 and CREB binding to the *il10* promoter (Figures 3C, 3D), and decreased the binding levels of NF-κB1 p50 with *il10* promoter (Figure 3B), which were consistent with previous findings that Akt phosphorylation can increase IL-10 production through either promoting transcriptional factors Ap1 and CREB binding with *il10* promote or reduction of NF-κB p105 stabilization [28]. However, down-regulation of p38 MAPK and ERK activation did not suppress the binding levels of NF-κB1 p50, Ap1 and CREB with *il10* promoter (Figures 3B-3D). In addition, ChIP assays were performed to examine the binding of Sp1 and STAT3 to the *il10* promoter in situ in early infected cells. PCV2 as well as PCV1 inoculation that did not show p38 MAPK and ERK activation, also failed to cause Sp1 and STAT3 binding to the *il10* promoter (Supplemental Figure 2). Thus, the ChIP assays for transcription factors binding to the *il10* promoter were accurate reflections of IL-10 transcriptional regulation. These data suggested that PI3K/Akt cooperated with NF-κB pathways promote IL-10 transcription via p50, CREB and Ap1 transcription factors in the earlier phase of PCV2 inoculation.

### PCV2 activates p38 MAPK and ERK pathways to further enhance IL-10 production in the later phase of infection

**Figure 4:**
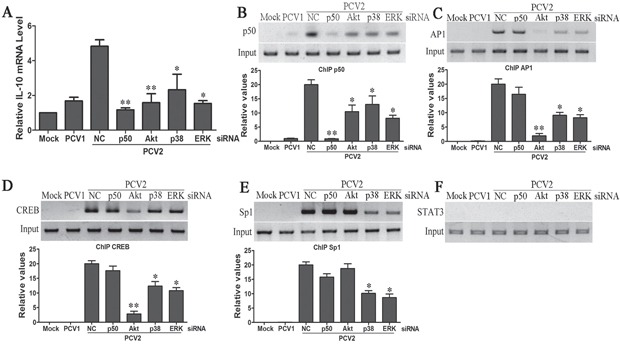
PCV2 activates p38 MAPK and ERK pathways to further enhance IL-10 production in the later phase of infection PAMs were transfected with the specific siRNAs of NF-κB, Akt, ERK or p38 MAPK, or negative control siRNA for 24 h, then cells were infected with 1 MOI of PCV2 for 12 h. The levels of IL-10 mRNA were detected by Q-PCR **A.** The binding activities of NF-κB p50 **B.** Ap1 **C.** CREB **D.** Sp1 **E.** and STAT3 **F.** were detected through ChIP assay. The relative binding levels of transcriptional factor with *il10* promoter were further exactly evaluated by Q-PCR (bottom panel in B, C, D, E). Mock and PCV1 inoculated cells were used as control in these assays. The results are mean ± SEM of 3 independent experiments. *P < 0.05, **P < 0.01 versus negative control (NC) siRNA-transfected cells.

In the later phase of PCV2 infection, Q-PCR detection of IL-10 transcriptional levels at 12 h p.i. showed that besides NF-κB p50 and Akt siRNAs, p38 MAPK and ERK siRNAs also markedly attenuated PCV2-induced IL-10 transcription (Figure 4A). ChIP assays showed that PCV2 also induced the efficient binding of NF-κB1 p50, Ap1, and CREB to the *il10* promoter (Figures 4B, 4C, 4D). Reduction of PI3K/Akt activation with Akt specific siRNA also decreased the binding levels of NF-κB1 p50 with *il10* promoter (Figure 4B), and prevented transcriptional factor Ap1 and CREB binding to the *il10* promoter (Figures 4C, 4D). Similarly, reduction of p38 MAPK or ERK with specific siRNAs decreased the binding levels of NF-κB1 p50, Ap1 and CREB with *il10* promoter (Figures 4B, 4C, 4D). Furthermore, ChIP assays examined the binding of Sp1 and STAT3 to the *il10* promoter in situ in late infected cells showed PCV2 caused Sp1 binding to the *il10* promoter, but did not cause STAT3 binding (Figures 4E, 4F). Reduction of p38 MAPK or ERK decreased transcriptional factor Sp1 binding to the *il10* promoter (Figure 4E). These data suggested that PCV2 activated p38 MAPK and ERK pathways to further enhance IL-10 production in the late phase of infection.

### PCV2 Cap protein is the key component in upregulating the expression of IL-10 in macrophages

**Figure 5:**
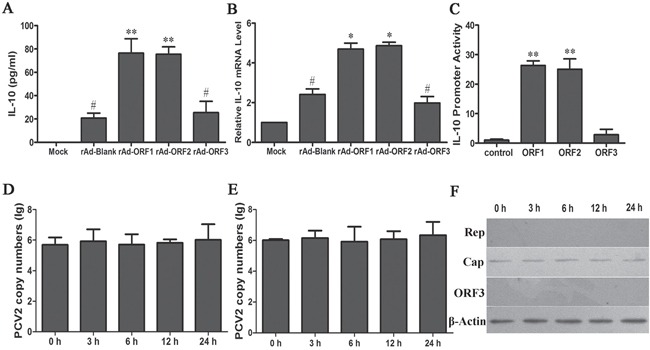
PCV2 Cap protein is a critical component for upregulating the expression of IL-10 in macrophages **A, B.** The roles of PCV2 Rep, Cap and ORF3 proteins in induction of IL-10 expression. The recombinant adenoviruses rAd-rep, rAd-cap and rAd-ORF3 or control rAd-Blank infected PAMs (1×10^6^ cells) at 100 MOI, and the IL-10 secretion and mRNA level were detected by ELISA (A) and Q-PCR (B), respectively. **C.** The activities of IL-10 promoter were measured in the PAMs co-transfected with Rep, Cap and ORF3 proteins expression vectors or blank vector (control) via Dual-Luciferase Reporter Assay System. **D, E.** The PCV2 copy numbers were analyzed by Q-PCR in primary PAMs (D) and 3D4/21 cells (E) inoculation with PCV2 at the indicated times. **F.** The expression of PCV2 Rep, Cap and ORF3 proteins were determined by western blotting in PCV2-inoculated PAMs. The data shown are representative of three independent experiments. *P < 0.05, **P < 0.01 versus rAd-blank-infected cells (A, B) or control (C). ^#^*P* < 0.05 versus Mock infection.

To further identify which component of PCV2 was involved in upregulation of IL-10 in macrophages, the three major ORFs of PCV2 were expressed by recombinant adenoviruses to identify their roles in induction of IL-10 expression. The results showed the IL-10 secretion and mRNA levels were both upregulated by PCV2 Rep and Cap proteins, but not by ORF3 (Figures 5A, 5B). *il10* promoter reporter activity assay also showed similar results as ELISA and Q-PCR detections (Figure 5C). However, previous studies found that PCV2 rarely replicates in monocytes or macrophages [29]. To determine whether PCV2 replicates in PAMs during IL-10 expression, primary PAMs and 3D4/21 cells were inoculated by 1 MOI of PCV2 and harvested to detect the copy numbers of PCV2 and the protein expression of Rep, Cap and ORF3 at 3 h, 6 h, 12 h and 24 h. Quantitative PCR results showed that PCV2 copy numbers did no significantly increase in 24 h post inoculation (Figures 5D, 5E). Meanwhile, only Cap proteins were detected at different times post-inoculation by western blotting (Figure 5F). These results suggested that Cap protein might be the critical component to upregulate or enhance IL-10 expression in PCV2-inoculated PAMs.

### PCV2 Cap activates PI3K/Akt, p38 MAPK, and ERK signaling pathways in macrophages

**Figure 6:**
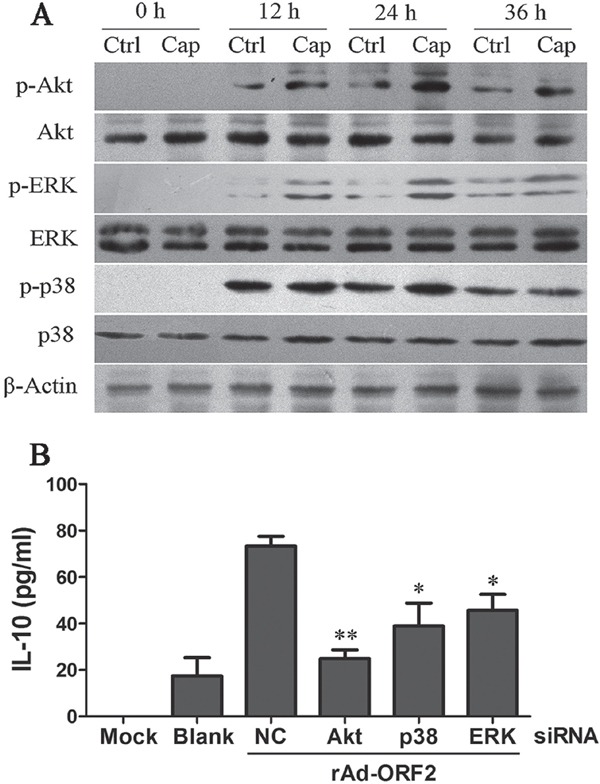
PCV2 Cap protein activates PI3K/Akt, ERK and p38 MAPK signaling pathways **A.** Either rAd-Cap (Cap) or negative control rAd-Blank (Ctrl) infected PAMs for 0 h, 12 h, 24 h and 36 h, Akt, ERK, p38 MAPK and their phosphorylation levels were analyzed by western blotting. **B.** The effects of PI3K/Akt, ERK or p38 MAPK signaling in PCV2 Cap-induced IL-10 secretion were measured at 24 h after rAd-ORF2 or rAd-Blank infection by using specific siRNAs of Akt, ERK or p38 MAPK. The data shown are representative of three independent experiments. *P < 0.05, **P < 0.01 versus negative control (NC) siRNA-transfected cells.

Since PCV2 Cap played a central role in PCV2- induced IL-10 expression, then we further determined whether Cap could also enhance the activation of these signaling pathways activated by PCV2 in recombinant adenoviruses rAd-Cap-infected PAMs. The results showed recombinant adenoviruses could partly activate these signaling pathways themselves. However, compared to empty recombinant adenoviruses (rAd-blank), rAd-ORF2 could significantly up-regulate p-Akt, p-p38 MAPK and p-ERK with no visible change of Akt, p38 MAPK and ERK (Figure 6A). Except that, there were no significant differences in IκB and JNK phosphorylation between rAd-blank and rAd-ORF2 (data no shown). Specific siRNAs were also employed to determine the roles of these signaling pathways in the expression of IL-10 induced by rAd-ORF2. Down-regulation of Akt, p38 MAPK, and ERK did inhibit rAd-ORF2-induced IL-10 production (Figure 6B). These results suggested that PCV2 Cap protein could up-regulate IL-10 expression though PI3K/Akt, p38 MAPK and ERK signaling pathways.

### PCV2 Cap protein binds with host gC1qR protein to promote IL-10 production

**Figure 7:**
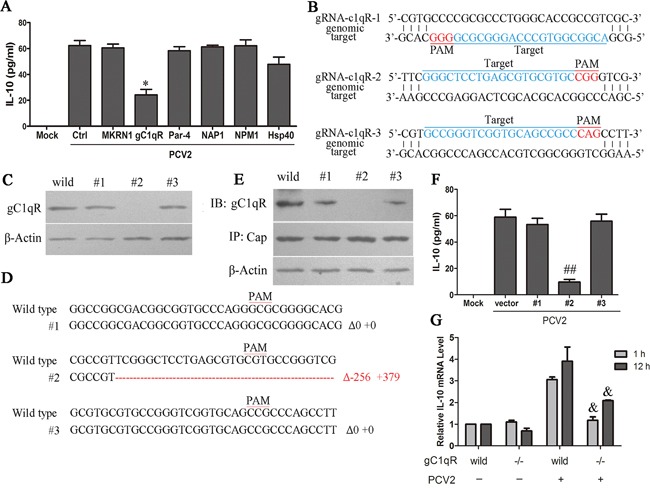
PCV2 Cap binds with gC1QR protein to promote IL-10 production **A.** The specific siRNAs of gC1QR, MKRN1, Par-4, NAP1, NPM1 and HSP40 were transfected into PAMs, then PCV2 infected the cells (1×10^6^ cells) for 24 h. The secretion of IL-10 in different siRNAs-transfected cells were measured by ELISA. **B.** Graphical representation of targeted porcine gC1QR loci. Targeted genomic loci are indicated in blue. Protospacer adjacent motifs (PAM) sequences are marked in red. **C.** Western blotting analysis of gC1QR proteins after CRISPR-Cas9 system targeting gC1QR locus infected PAMs. #1, #2 and #3 indicated the cells infected with lentiviruses containing gRNA-c1qR-1, gRNA-c1qR-2 and gRNA-c1qR-3, respectively. **D.** Sanger sequencing of genomic PCR products encompassing the genomic-editing site from cells #1, #2 and #3. The WT reference sequence is shown on the top. The bottom is targeted sequence with both deletions and insertions. The sizes of insertion (+), deletion (Δ) are presented on the right of each allele. Red dashes: deleted and insertion bases; **E.** Immunoprecipitation analysis of the interaction of Cap with gC1QR after CRISPR-Cas9 system targeting gC1QR locus in PAMs. **F.** The IL-10 secretion were measured in wild-type and #1, #2 and #3 cells 24 h after PCV2 inoculation. **G.** The IL-10 mRNA levels were measured in wild-type and gC1QR−/− cells (#2) 1h and 24 h after PCV2 inoculation. The results are mean ± SEM of 3 independent experiments. *P < 0.05 versus control siRNA-transfected cells (Ctrl).^##^*P* < 0.01 versus the cells infected with control lentiviruses without gRNA (vector). ^&^*P* < 0.05 versus wild-type cells (wild) at same time.

Previous studies have found six binding proteins of PCV2 Cap, including makorin-1 RING zinc finger protein (MKRN1), receptor protein for the globular heads of complement component C1q (gC1qR), prostate apoptosis response-4 (Par-4), nucleosome assembly protein-1 (NAP1), nucleophosmin-1 (NPM1) and heat shock protein 40 (HSP40). To investigate which are involved in IL-10 induction, siRNAs specific for MKRN1, gC1qR, Par-4, NAP1, NPM1 and HSP40 were transfected into cells before PCV2 inoculation, respectively, and gene silencing was confirmed by western blotting (Supplemental Figure 3). Down regulation of gC1qR decreased approximately 2/3 of IL-10 production in PCV2 inoculated cells, while others did not show so significant effect on IL-10 production (Figure 7A). gC1qR plays an important role in the course of infection and pathogenesis of some viruses through interaction with viral proteins [30, 31]. Since siRNA was not able to completely abolish the presence of gC1qR in cells, CRISPR/Cas9 system, a novel genome editor tool, was used to generate specific gC1qR gene knockout macrophages in this study. Three gRNAs (gRNA-c1qR-1, gRNA-c1qR-2, gRNA-c1qR-3) were designed to target three different targeted sites of *gC1qR* gene (Figure 7B), and then three cell clones (#1, #2, #3) were isolated and identified. Western blotting analysis showed the gC1qR protein could not be detected in one of the three cell clones (#2), but the other two (#1, #3) still expressed gC1qR protein (Figure 7C). The sequencing showed the targeted sequences were deleted and inserted another sequence in the cell #2 clone that gC1qR protein could not be detected (Figure 7D). In the wild-type cells and the cell clones that gC1qR were not knockout (#1 and #3), immunoprecipitation results proved that Cap did bind with gC1qR protein, whereas this interactions were not detected in the gC1qR knockout cell clone (#2) after PCV2 inoculation (Figure 7E).

Next, PCV2 inoculated the gC1qR knockout and wild-type cells at 1 MOI for 24 h, and the secreted IL-10 were detected by ELISA. Results showed that the gC1qR knockout cells produced less IL-10 in the supernatants compared to the wild-type cells, whereas the cell clones (#1 and #3) that gC1qR were not knockout produced IL-10 as well as the wild-type cells (Figure 7F). Consistently, the IL-10 mRNA levels were also significantly decreased in the gC1qR knockout cells when infected by 1 MOI PCV2 at both 1 h and 12 h (Figure 7G). These results suggested that PCV2 Cap protein combined to host gC1qR to promote IL-10 production at the both early and late phase of infection, and deletion of gC1qR almost completely abolished PCV2-indcued IL-10 expression.

### gC1qR medicates PCV2-induced PI3K/Akt and p38 MAPK signaling activation to promote IL-10 induction in macrophage

**Figure 8:**
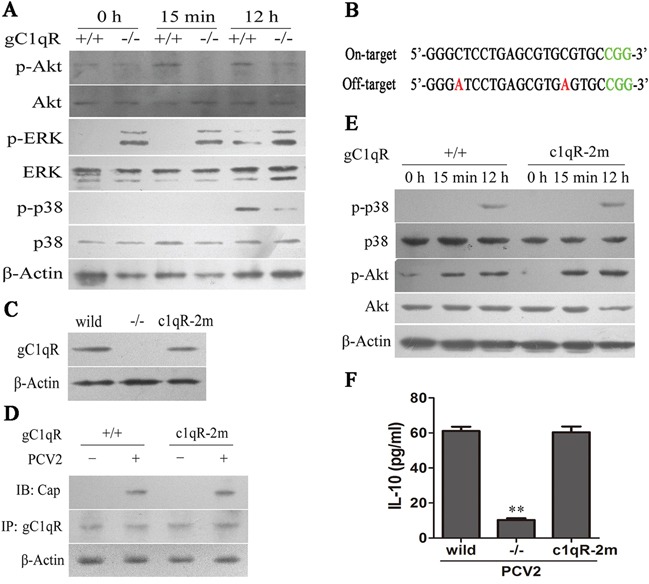
PCV2 binding with gC1QR to activate PI3K/Akt and P38 MAPK **A.** Western blot analysis of the phosphorylation levels of Akt, ERK1/2 and p38 MAPK in wild-type and gC1QR−/− cells inoculated with PCV2 for indicated times. **B.** Mutated gRNA (gRNA-c1qR-2m) sequences for off-targeting gC1QR locus and corresponding protospacer adjacent motifs (PAM). Red bases: mutations; green bases: PAM. **C.** Western blotting analysis of gC1QR expression in the cells infected lentivirus containing gRNA-c1qR-2 or gRNA-c1qR-2m as well as wild-type cells. **D.** Immunoprecipitation analysis of the interaction of Cap with gC1QR in wild-type cells and the cells infected lentivirus containing gRNA-c1qR-2m after PCV2 inoculation **E.** Western blot analysis of Akt phosphorylation in gRNA-c1qR-2m lentivirus infected cells and wild-type cells after PCV2 inoculation. **F.** ELISA detection of PCV2-induced IL-10 production in wild-type cells, gRNA-c1qR-2 lentivirus-infected cells and gRNA-c1qR-2m lentivirus-infected cells (1×10^6^ cells). The data shown are representative of three independent experiments. The results are mean ± SEM of 3 independent experiments. **P < 0.01 versus PCV2-inoculated wild-type cells.

The data from the current study demonstrated that PCV2 Cap activated PI3K/Akt, p38 MAPK, and ERK signaling pathways, but only PI3K/Akt signaling was involved IL-10 induction in the both early and late phase of PCV2 inoculation in macrophage, while PCV2 Cap protein binding with host gC1qR to induce IL-10 production at the both early and late phase of infection. Thus, we further assessed the roles of gC1qR in the activation of PI3K/Akt, p38 MAPK, and ERK signaling pathways induced by PCV2. Compared with gC1qR wild-type cells (gC1qR+/+), gC1qR knockout cells (gC1qR−/−) did not appear significant phosphorylation of Akt at 15 min and 12 hours post inoculation, while the phosphorylation of p38 MAPK was reduced significantly at 12 h p.i. (Figure 8A). However, the phosphorylation of ERK1/2 appeared to be increased in gC1qR−/− cells spontaneously, while ERK1/2 was not significantly decreased in gC1qR−/− cells compared to gC1qR+/+ cells (Figure 8A). These results demonstrated that PCV2-induced PI3K/Akt and p38 MAPK activation are dependent on gC1qR, while ERK signaling pathway activation is independent on gC1qR.

To eliminate the possibility of off-target effects for the CRISPR/Cas9 system sgRNA, we introduced two mutation sites into gRNA-c1qR-2 (Figure 8B) to test whether the mutated gRNA could affect the expression of gC1qR. Western blotting showed that gC1qR protein levels were not affected in the cell clone that infected with mutated gRNA-c1qR-2 lentivirus (gRNA-c1qR-2m) (Figure 8C). The binding of gC1qR with Cap, and Akt and p38 phosphorylation induced by PCV2 inoculation also kept same levels as in gC1qR+/+ cells (Figures 8D, E). Consistently, the PCV2-induced IL-10 production did not change in the cell clone that infected with mutated gRNA-c1qR-2 lentivirus (gRNA-c1qR-2m) (Figure 8F). Taken together, these results further confirmed that gC1qR medicated PCV2-induced PI3K/Akt and p38 MAPK activation to promote IL-10 induction by interaction with Cap in macrophages.

## DISCUSSION

**Figure 9:**
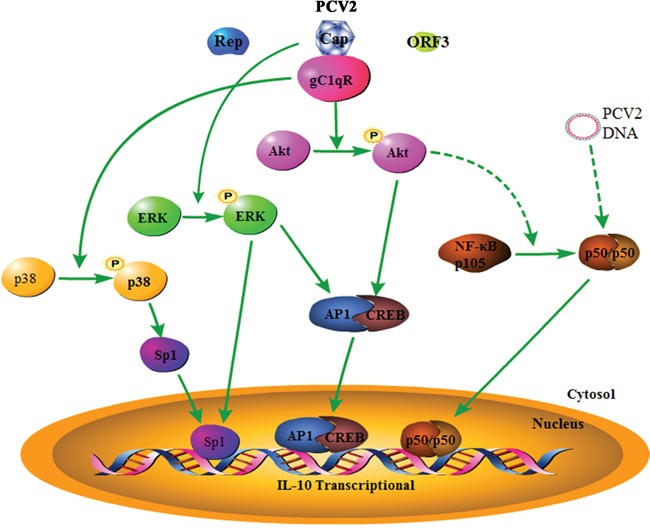
Model of PCV2 inoculation regulates IL-10 production in porcine alveolar macrophages PCV2 inoculation induces the phosphorylation of Akt, p38 MAPK and ERK through viral Cap protein, activates corresponding PI3K/Akt, p38 MAPK and ERK pathways, which in turn together with NF-κB pathway to promote transcriptional factor Ap1, CREB, Sp1 and NF-κB1 p50 translocation into nuclear and binding with *il10* promoter, resulting in IL-10 expression in macrophages. In this process, gC1qR mediates PCV2-induced PI3K/Akt and p38 MAPK activation by interaction with PCV2 Cap, which plays a pivotal role in promoting IL-10 induction, whereas PCV2 Rep and ORF3 are not involved in the induction of IL-10 in PCV2-inoculated macrophages. The solid lines based on our results. The dashed lines based on other reports or our results that were not shown here.

Numerous viruses have been verified to induce the production of IL-10 with an enhancement of infection by suppression of immune functions [5, 6]. Previous studies dealing with the cytokine patterns in tissues or PBMC of PCV2-induced PMWS pigs have a common finding is elevated level of IL-10 expression [8, 15]. As first defensive line of host, alveolar macrophage plays an important role in anti-virus infection [32]. PCV2 was found entering PAM without detectable replication, however, the virus increased the secretion of tumor necrosis factor-α (TNF-α) and interleukin-8 (IL-8), as well as up-regulation of mRNA of macrophage-derived chemotactic factor-II (AMCF-II), granulocyte colony-stimulating factor (G-CSF) and monocyte chemotactic protein-1 (MCP-1) [33]. In this study, our results demonstrated that PCV2, but not non-pathogenic PCV1, also greatly induced IL-10 production in PAMs. Furthermore, we found that IL-10 was induced to express upon initial PCV2 inoculation in PAMs by activation of NF-κB pathway and PI3K/Akt pathway, and further enhanced in latter phase of infection by activation of ERK and p38 MAPK pathways. In the whole process of IL-10 induction by PCV2, the interaction of PCV2 Cap and Cap-binding protein (gC1qR) played a pivotal role through activation of PI3K/Akt and p38 MAPK pathways to promote transcriptional factors Ap1, CREB and Sp1 binding with *il10* promoter and increased the levels of NF-κB p50 (Figure 9). The results present in this study systematically demonstrated how PCV2 induces IL-10 expression in PAMs, and will contribute to explain why PCV2 can induce immune suppression and cause PMWS whereas PCV1 can not.

NF-κB signaling pathway is found to be vital in various viruses induced IL-10 production [5, 34, 35]. The NF-κB p50 homodimers have been identified as the key transcriptional factor in regulation of IL-10 expression [2, 3]. Previous studies showed that PCV2 infection activated NF-κB pathway in PK-15 cells and liver cells of PCV2 infected pigs [36, 37]. In this study, we found that PCV2 inoculation led to IκB phosphorylation and NF-κB p50 subunit translocation to the nucleus of PAMs, while p65 did not show visible changes. These results hinted that PCV2 inoculation promoted NF-κB p50/p50 translocation into the nucleus. In most of virus-infected cells, NF-κB pathway were usually activated by viral DNA or RNA stimulation [38]. We have also found that both PCV1 and PCV2 DNA could induce the activation of NF-κB pathway, but that were not enough to be able to induce detectable IL-10 expression (data not shown). So we considered the PCV2 DNA was the key component to activate NF-κB signaling in PCV2-inoculated cells. The results of ChIP assay confirmed that NF-κB1 p50 subunit did bind to the *il10* promoter in both earlier and later phases of IL-10 transcription. However, as transcription regulation component, only NF-κB1 p50 subunit could not effectively activate the transcription of *il10* gene in PCV2-inoculated PAMs, which had been confirmed in this study.

The production of IL-10 by macrophages is also regulated by PI3K/Akt pathway [4]. PI3K/Akt pathway has been showed to be activated in PCV2 infected PK-15 cells and involved in regulation of the apoptosis of the infected cells and virus growth [25]. In present study, PI3K/Akt pathway was found to be activated in PCV2 infected PAMs. The transcriptional factors AP1 and CREB were also found binding to *il10* promoter in both earlier and later phases of PCV2 inoculation. However, we did not found the activation of PI3K/Akt pathway in PCV1 infected PAMs. In addition, in variant viruses induction of IL-10, different MAPK pathways are employed [5, 34, 39]. Although JNK signaling has been reported to be involved in PCV2-induced cell apoptosis in infected PK-15 together with p38 MAPK signaling [26], JNK was not activated in PCV2-inoculated PAMs. ERK and p38 MAPK were found to be activated to enhance IL-10 expression in the later phase of PCV2 inoculation. Transcription factor Sp1 regulated by ERK and p38 MAPK was found binding to *il10* promoter in later phase. These results suggested that in PCV2-inoculated PAMs, the activation of PI3K/Akt, ERK and p38 MAPK signaling pathways are more critical for the high expression of IL-10. Additionally, we noted that the inhibitors or siRNAs of NF-κB and Akt could reverse the up-regulation of IL-10 in both earlier and later phases through decreasing the binding of p50, AP1 and CREB to *il10* promoter, whereas ERK and p38 MAPK inhibitors or siRNAs just partly prevented the IL-10 up-regulation in later phase via suppressing the binding of Sp1 to *il10* promoter, suggesting that NF-κB and PI3K/Akt were involved in the regulation of IL-10 expression in both the earlier and later phase, while ERK and p38 MAPK signaling just participated in the later phase of PCV2-induced IL-10. Interestingly, blocking ERK and p38 MAPK affected all transcriptional factors. For the possible reason, p38 and ERK are reported to regulate IL-10 production in macrophages by AP1, Sp1, and CREB [2, 40]. Furthermore, ERK is also demonstrated to regulate p50 homodimer DNA binding activity [41], while p38 MAPK is reported to control NF-κB transcriptional activation [42]. Taken together, our results demonstrated that for PCV2-induced IL-10 production, NF-κB, PI3K/Akt, ERK and p38 MAPK signaling pathways are all involved, while NF-κB and PI3K/Akt signalings participate in the whole process, and ERK and p38 MAPK signalings just further enhance IL-10 production in the later phase of PCV2 inoculation. However, a question need to be further explored is how PCV2 control these processes.

PCV2 contains 11 predicted ORFs [16]. ORF1, ORF2 and ORF3 encoded proteins have been well confirmed. Previous studies suggested that ORF2 and ORF3 encoded proteins play important roles in the pathogenesis of PCV2. Mutation or nucleotide deficiency of the ORF2 or ORF3 gene could alter the virulence of PCV2 [18, 43]. In this study, all the three proteins were tested to identify whether they could promote IL-10 production or not. Both Rep and Cap were proved to promote IL-10 production, while ORF3 did not. Previous studies showed PCV2 might replicate in some of the macrophage-like cells, but the replication seemed to be very low [29, 33]. We further checked the copy numbers of PCV2 and the expression of ORF1, ORF2 and ORF3 in the PAMs in this study, but replication was not obvious in the process. In addition, we found that the genome DNA of both PCV1 and PCV2 had the ability to induce the transcription of *il10* gene by reporter gene assay, and did not show significant difference between them in induction capability (data not shown). In further studies, we found that both empty recombinant adenoviruses (rAd-blank) and recombinant adenoviruses expressing Cap protein (rAd-ORF2) could activated NF-κB signaling, and Cap expression did not further increased the phosphorylation level of IκB (data not shown), suggesting Cap protein were not able to activate NF-κB signaling, NF-κB signaling were activated by adenoviruses as previous studies [44]. However, Cap protein could activate PI3K/Akt, p38 MAPK and ERK pathways as shown in results. Taken together, our data in this study demonstrated that both Cap protein and virus DNA were required for the high expression of IL-10 in PCV2-inoculated cells, and that Cap protein was major component of PCV2 in enhancing the expression of IL-10 in PCV2-inoculated cells through activation of PI3K/Akt, p38 MAPK and ERK pathways. Therefore, the results presented in here also suggested that even though Cap protein was the critical component for PCV2-indcued IL-10 in alveolar macrophages, only Cap protein were not able to induce IL-10 expression, and suggested that the difference of Cap proteins between PCV1 and PCV2 might be a major reason for why PCV2 can induce immune suppression and cause PMWS whereas PCV1 can not.

Previous studies found that there were six possible Cap-binding proteins in PCV2-infected cells (MKRN1, gC1qR, Par-4, NAP1, NPM1 and Hsp40) [24]. In this study, the results showed that gC1qR was probably the major protein employed in PCV2-induced IL-10 production. To further identify the roles of gC1qR in PCV2-induced IL-10 production, we employed CRISPR/Cas9 system to generate a gC1qR knockout PAMs. Upon PCV2 inoculation, IL-10 production significantly decreased in the gC1qR knockout PAMs at both protein and mRNA levels in both earlier and later phases. The phosphorylation of Akt were blocked at the 15 min and 12 h p.i., and p38 MAPK phosphorylation significantly reduced at 12 h p.i., while IκB activation did not changed significantly in the gC1qR knockout cells. These results demonstrated that the activation of PI3K/Akt and p38 MAPK are depending on the binding of PCV2 Cap with host gC1qR. However, which Cap binding proteins are involved in PCV2-induced ERK signaling activation need further study. Ongoing work in our laboratory is addressing these issues.

In summary, the present data in this study provide certain evidences for the roles of gC1qR, PI3K/Akt and p38 MAPK pathways in PCV2 induction of IL-10 production, and systematically demonstrate the regulation roles and patterns of different signaling pathways in PCV2-induced IL-10 expression in macrophage. These finding should help us to further understand why PCV2 can induce immune suppression and cause PMWS whereas PCV1 can not.

## MATERIALS AND METHODS

### Ethics statement

All animal experiments were approved by the Institutional Animal Care and Use Committee (IACUC) of Forth Military Medical University (permit number: 13024), and were performed according to the Animal Ethics Procedures and Guidelines of the People's Republic of China. No other specific permissions were required for these activities. This study did not involve endangered or protected species.

### Cell culture and virus preparation

PK-15 cells and porcine alveolar macrophages cell line 3D4/21 (CRL-2843) were purchased from ATCC. The primary porcine alveolar macrophages were obtained from the lungs of SPF piglets as previously described [33]. PK-15 cells were maintained in Dulbecco's minimum essential medium (DMEM) supplemented with 10% heat-inactivated fetal bovine serum (FBS). 3D4/21 and primary PAMs were cultured in RPMI 1640 medium with 10% heat-inactivated FBS, sodium pyruvate, nonessential amino acids, 100 U/ml penicillin and 0.1 mg/ml streptomycin.

PCV1 (AY193712) and PCV2 (EU366323) were both isolated and stocked in our laboratory [45]. And the PCV viruses were propagated in PK-15 cells. The copy numbers of the viruses were measured by Q-PCR. The polyclonal antibodies against PCV2 Rep, Cap and ORF3 proteins were prepared and kept in our laboratory [46].

### ELISA

PAMs were adhered to 6 well plates followed by PCV infection at 1 MOI. The supernatants were harvested to detect IL-10 secretion by enzyme-linked immunosorbent assay (ELISA) kit (R&D) according to the manufacturer's instructions.

### Quantitative PCR

The diluted plasmids containing PCV1 and PCV2 whole genomes were used as templates to draw standard curve. Virus DNA was isolated by proteinase K and SDS to determine the copy numbers of the harvested viruses. The primers were: PCV-F: AGTACCGGGAGTGGTAGGAG; PCV-R: GTTGAATTCTGGCCCTGCTC.

Total cellular RNA was isolated by TRIZOL according to the manufactory's instructions. Reverse transcription was performed using M-MLV reverse transcriptase (Invitrogen) with Random primer. IL-10 mRNA level was analyzed by SYBR-green based Q-PCR using a Bio-Rad IQ5 Real-Time PCR System. IL-10 mRNA were normalized by comparing to β-actin and expressed relative to mock control. Primer sequences were: IL-10-F: AATCTGCTCCAAGGTTCCCG; IL-10-R: TGAACACCATAGGGCACACC; β-actin-F: GGACTTCGAGCAGGAGATGG; β-actin-R: AGGAAG GAGGGCTGGAAGAG.

### Luciferase reporter assays

Porcine *il10* promoter sequence was amplified and cloned into pGL3 basic vector (Promega) according to Rong Quan, *et al*. [47]. 3D4/21 cells were co-transfected with a mixture of pGL-IL-10 plasmid and pRL-TK renilla luciferase plasmid using Lipofectamine 2000 (Invitrogen). The PCV viruses were infected to the cells for 24 h. At 24 h post infection, luciferase activities were determined via Dual-Luciferase Reporter Assay System (Promega) according to the manufacturer's instructions.

### Inhibition of signaling pathways

For specific inhibitors treated cells, PAM cells were pre-incubated with DMSO, or NF-κB inhibitor BAY11-7082 (Merck), PI3K/Akt inhibitor LY294002 (Merck), ERK1/2 MAPK inhibitor PD98059 (Merck), p38 MAPK inhibitor SB203580 (Merck) and JNK inhibitor SP6000125 (Sigma) for 1 h. For siRNA treated cells, PAM cells were transfected with specific siRNAs (Supplemental Table 1) or negative control siRNA using Lipofectamine 2000 for 24 h. Then cells were infected with 1 MOI of PCV2 for 1 h, 12 h or 24 h. Cells were harvested to extract total RNA and proteins. IL-10 mRNA and protein levels were evaluated by Q-PCR and ELISA, respectively. The inhibition of signaling pathways were checked by western blotting.

### Construction of PCV protein-encoding plasmids and recombinant adenoviruses

Genes encoding viral proteins were amplified from PCV2 genomes and cloned into pCI-neo between *Xho* I and *Not* I sites or recombinant shuttle vector pShuttle-CMV between *Sal* I and *Not* I. The confirmed pShuttle-ORFs were recombinant with the bone vector pAdeasy-1 in *E. coli* BJ5183, and the recombined plasmids were transfected into 293A cells after linearization to generate recombinant adenoviruses according to the manufactory's instruction. The recombinant adenoviruses infected 3D4/21 cells at 100 MOI for further analysis.

### Western blotting analysis

The cells were lysed in RIPA with 1 mM PMSF and protease inhibitors (Sigma) for 15 min on ice. The cytosol and nuclear fraction were isolated according to the manufactory's instruction (Thermo). Similar amounts of protein from each extract were subjected to SDS-PAGE analysis and transferred to polyvinyl difluoride (PVDF) membranes (Millipore). After blocking for 1 h with blocking buffer (5% nonfat milk and 0.1% Tween-20 in PBS), the membranes were incubated with the following primary antibodies at 4°C overnight: anti-phospho-p38 MAPK, anti-p38 MAPK, anti-phospho-JNK, anti-JNK (Santa Cruz), anti-phospho-ERK, anti-ERK, anti-phospho-Akt, anti-Akt, anti-phospho-IκBα, anti-IκBα, anti-p50/p105, anti-p65, anti-Histone H3 (Cell Signaling Technology), anti-gC1qR (Hycult biotech) and anti-β-actin (Tianjin Sungene Biotech) antibodies. HRP-conjugated anti-mouse IgG or anti-rabbit IgG (Santa Cruz) were used as secondary antibodies. And ECL (Bio-Rad) was used for chemiluminescent detection according to the manufacturer's instructions.

### Chromatin immunoprecipitation (ChIP)

The ChIP assays were proceeded following the Cold Spring Harbor Protocols [48]. Briefly, the cells were cross-linked by formaldehyde before lysis for nuclear. The nuclear were then further lysed by nuclei lysis buffer for the chromatin and proceeded with sonication. The chromatin were quantified and divided into 100 μg per antibody. NF-κB p50, c-jun, CREB, Sp1 and STAT3 monoclonal antibodies (Cell Signaling Technology) and irrelevant antibodies were added to the chromatin samples overnight at 4°C following preclear by protein A(G)-agarose/salmon sperm DNA beads. Protein A(G)-agarose/salmon sperm DNA beads were added into the samples again to bind to the antibodies-chromatin compound. Then the compound were digested by proteinase K, and extracted by Phenol:Chloroform to purify nucleic acids. The nucleic acids were resuspended in H_2_O and analyze by PCR, and further quantified by Q-PCR. The specific primers for PCR and Q-PCR: AP1-F: CCAGCTGTGGAAGCTCACAA; AP1-R: GGAACAACGGGCCATGCTTA; NF-F: TTGGAGAGGTCTAGGGAAGGG; NF-R: AGAGCT GTGCCTTCTTCGTT; CREB-F: CAGCAAGGAGAA CCCTTGAGT; CREB-R: AGGTCACAATGACG TGGACA; SP1-F: ACACGTGAATGGAACCCACA; SP1-R: GAGGCTACCTCTCTCCCCTT; STAT3-F: TGCAAAGTTGGAGAGGTCTAGG; STAT3-R: AGTGAGGCCTCCCCATTCAT; NS-F: GTAAGCATGGCCCGTTGTTC; NS-R: GTTTTCTGTTCCAAGCCCGC.

### Immunoprecipitation (IP)

The pCI-ORF2 plasmids were transfected into wild-type and gC1qR knockout cells. The cells were harvested and lysed by RIPA buffer on ice for 15 min. The supernatant were pre-cleared to reduce non-specific binding to the Protein A/G agarose beads. 1 μg rabbit anti PCV2 Cap polyclonal antibodies or mouse anti gC1qR monoclonal antibodies were added into the cell lysates and incubated with gentle rocking overnight at 4°C. Protein A/G agarose beads were added and incubated, then centrifuged, and the pellets were washed five times with cell lysis buffer. The pellets were resuspended with SDS sample buffer to perform western blotting analysis.

### Generation of gC1qR knockout PAMs

Three pairs of oligos were designed based on the porcine gC1qR sequence (KM068128). The oligos were cloned into the BsmB I sites of lentiCRISPRv2 plasmid (Addgene). And the recombinant plasmids were transfected into 293T cells to obtain recombinant lentivirus. The recombinant lentiviruses were used to infect PAMs, respectively. 24 hours later, 5 μg/ml puromycin were added into the cell cultures to select the knockout cells. The selected cells were checked by western blotting and sequencing.

### Statistical analysis

All experiments were performed at least three times, and results are representative of three of independent experiments. Data were presented as means ± SEM (SD). Multiple group data were analyzed by ANOVA and Bonferroni post-hoc test, while comparisons between 2 groups were performed by unpaired Student's t test. Statistical significance was defined as P < 0.05.

## SUPPLEMENTARY FIGURES AND TABLE


