# Supplementary material for: Porcine circovirus type 2 activates PI3K/Akt and p38 MAPK pathways to promote interleukin-10 production in macrophages via Cap interaction of gC1qR
Source: Oncotarget. 2016 Feb 13;7(14):17492–507. doi: 10.18632/oncotarget.7362 (PMC4951228; doi:10.18632/oncotarget.7362)
Supplement: Supplementary file 1 [file oncotarget-07-17492-s001.pdf]

SUPPLEMENTARY FIGURES AND TABLE

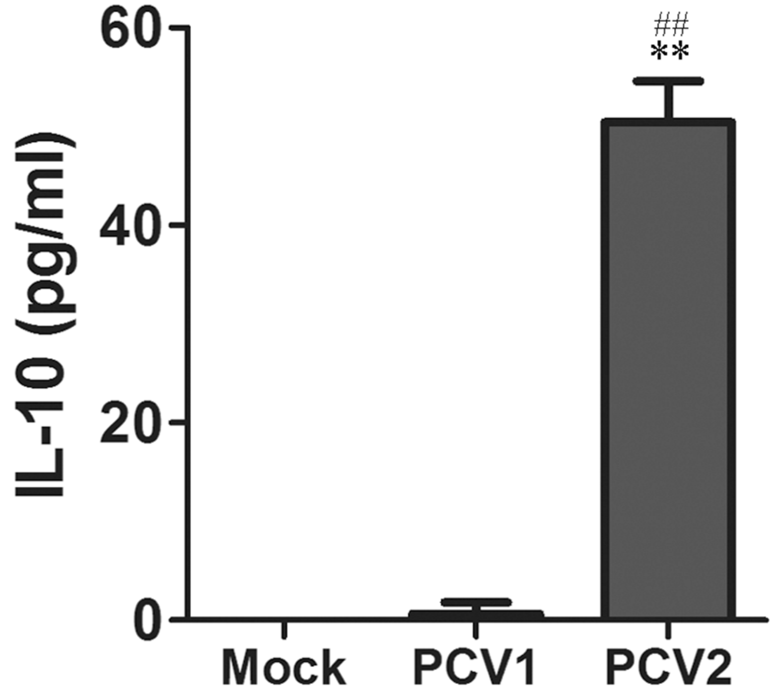

Supplementary Figure 1: PCV2 inoculation induces IL-10 production in 3D4/21 cells. 1 MOI of PCV1 or PCV2 inoculated porcine alveolar macrophages cell line (3D4/21) cells for 24 h, and then the secretion of IL-10 were detected by ELISA (1×10^6 cells). The results are mean ±SEM of 3 independent experiments. $^{**}P < 0.01$ versus Mock inoculated cells. $^{\#\#}P < 0.01$ versus PCV1 inoculation.

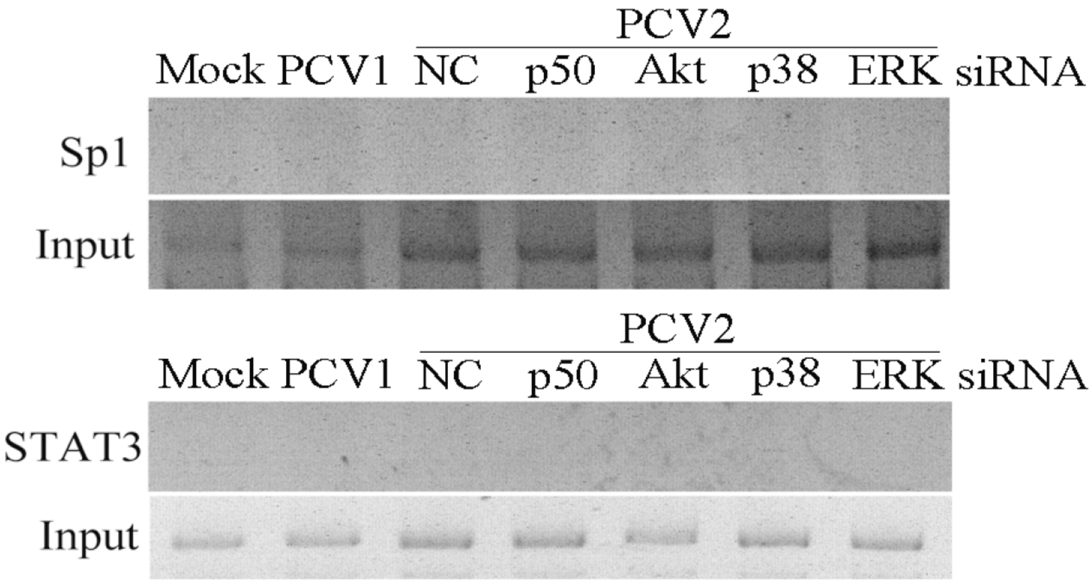

Supplementary Figure 2: Sp1 and STAT3 did not bind to *il10* promoter in the earlier phase of infection. The ERK and p38 MAPK specific siRNAs transfected PAMs for 24 h, followed PCV2 inoculation for another 1 h. The nuclear fraction of the cells was isolated, and the binding activities of Sp1 and STAT3 were tested by ChIP assay. Mock and PCV1 inoculated cells were used as control in these assays. The data shown are representative of three independent experiments.

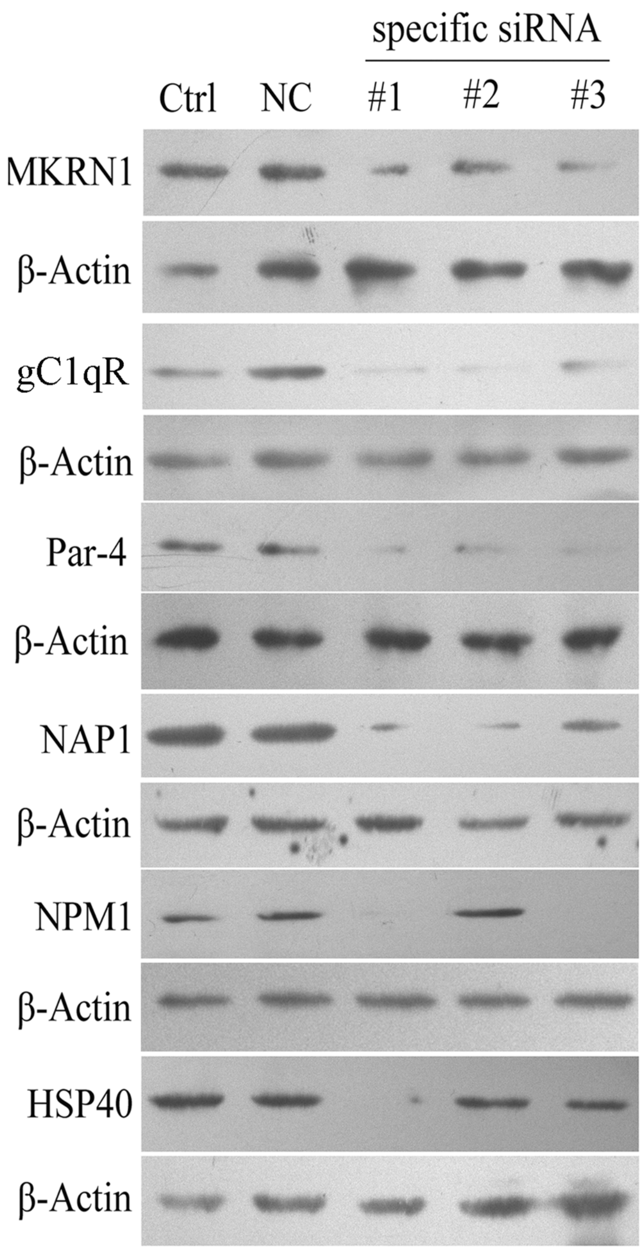

Supplementary Figure 3: Comparing the knockdown efficiency of isoform specific siRNA for Gene silencing of MKRN1, gC1QR, Par-4, NAP1, NPM1 and HSP40. Three specific siRNAs to MKRN1, gC1QR, Par-4, NAP1, NPM1 and HSP40 were transfected to cells for 48 h, the knockdown efficiency of each gene was detected by western blotting.

Supplementary Table 1: siRNA sequences for target genes

Target gene	Accession number	Sequences
NF-κB p50	KC316024.1	AAGGAGATCATCCGTCAGGCA
		AAGCACGAATGACAGAGGCAT
		AAGGAGGAGAATTACAGGTTC
Akt	NM_001159776.1	AACGAGGCGAGTACATCAAGA
		AAGAACGACGGCACCTTCATC
		AAGGAGATCATGCAGCACCGT
p38	XM_001929490.5	AAGCTATCCAGACCATTTCAA
		AAGAAGAGATGGAGTCTTGAG
		AAGGTGTCTCCATTTCTGTCA
ERK	NM_001198922.1	AAGCTCTTGAAGACGCAGCAC
		AAGCTCTGGATTTACTGGACA
		AAGCACCATTCAAGTTTGACA
MKRN1	XM_013983674.1	AAGTCTCTCAGCAGTAGTTGG
		AAGTGGAGGAGTGCTAAGCAG
		AAGCGGAGTCAAGAAATTCAA
gC1qR	AK236153.1	AAGATACAGAAGCATAAGTCT
		AAATGCTGCAACTGCTACGCT
		AACAGGAGCCTGAACTGACGT
Par-4	XR_001303231.1	AAGATGCAATTACACAACAGA
		AAGTTCCTACCTGCTGCAAGA
		AACGAGAAGATGCAATTACAC
NAP1	NM_001285976.1	AAGACGTACAAGATGAAGTCA
		AAGCTCACAGACCAGGTGATG
		AAGTGTCTGATTGATGCGGCT
NPM1	XM_003359819.3	AAGGCAGTCCAATCAAAGTAA
		AAGGATGAGTTGCACATTGTA
		AAGGACCTAGTTCTGTAGAAG
Hsp40	XM_003131409.4	AAGGCCTGCATTGCTTGCAGA
		AAGCTAGCATATGAACTGTAC
		AAGCCATAGATATGTGTCCTA
